# Metronomics for thymic carcinoma

**DOI:** 10.3332/ecancer.2014.494

**Published:** 2014-12-18

**Authors:** Jacobo Muñoz del Toro, Patricia Cortez Castedo, Alfonso Cortés Salgado, M Eugenia Olmedo García, Pilar Garrido López

**Affiliations:** Medical Oncology Department, Hospital Universitario Ramón y Cajal, Ctra de Colmenar Viejo km 9, Madrid 28034, Spain

**Keywords:** metronomics, recurrent thymic carcinoma, treatment

## Abstract

Although thymomas are the most frequent primary tumours of the anterior mediastinum, thymic carcinoma is very infrequent and more aggressive. Combination chemotherapy is the first-line treatment for the advanced stages, but because of the lack of evidence from randomised trials, the management of the successive lines is a challenging field. We report a partial radiological response in the seventh line of a thymic carcinoma stage IV with an oral regimen.

## Introduction

Thymic epithelial tumours are divided into two categories, thymomas and thymic carcinomas. Although thymic tumours are rare (0.2–1.5% of all malignancies; 1.3 cases per million population in the US [1]), thymomas are the most frequent primary tumours of the anterior mediastinum [2]. On the other hand, thymic carcinoma is responsible for less than 1% of thymic neoplasms. It is more aggressive than thymoma and often metastasizes [3–4]. The differential diagnosis between thymoma and thymic carcinoma is complex and mainly based on histological and immunohistochemical differences [5]. We report the case of a patient with an initial stage IVA Masaoka-Koga staging system thymic carcinoma lasting 11 years who achieved a partial response by using a metronomic approach in his seventh line of treatment.

## Case report

The patient was a 41-year-old male with no relevant medical history who in October 2001 was diagnosed by biopsy of a stage IVA (pleural involvement) thymic carcinoma. The patient received first-line chemotherapy in other hospital. The selected regimen was cisplatin, adriamycin, and cyclophosphamide (CAP) followed by etoposide, ifosfamide, and cisplatin (VIP) completing six cycles, achieving a partial response and passing to monitoring. After a progression-free interval of 4 years, a relapse was diagnosed including pulmonary metastasis together with positivity for somatostatin receptor scintigraphy. The patient was staged as stage IVB, and for the next 7 years, the patient received several treatment lines successively, including lanreotide and steroids, gefitinib, paclitaxel, carboplatin, etoposide, sunitinib as well as palliative radiotherapy. The patient achieved prolonged stabilizations as best response lasting less than 1 year each.

**Figure 1.**
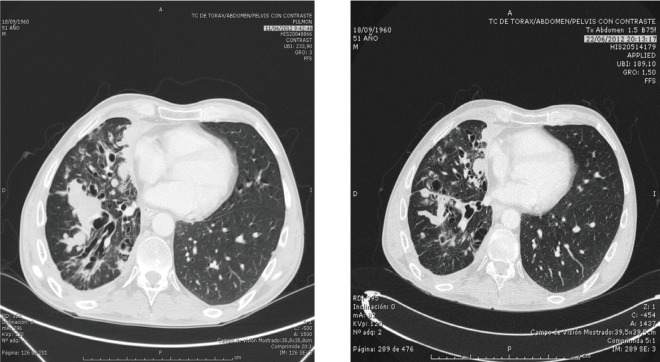
The response after metronomic treatment.

On February 2012, the patient was admitted to our hospital, where a new line of treatment was offered. The regimen included continuous oral cyclophosphamide (50 mg/day) and prednisone (100 mg per day) plus monthly intramuscular lanreotide at a dose of 60 mg. After two cycles, a partial response was visible on the CT scan (Figure 1). The patient continued with the same regimen except cyclophosphamide that had to be abandoned after the fourth cycle due to a progressive deterioration of the renal function. Additionally, the patient had anaemia grade 2 and fatigue grade 1 as more relevant toxicity. With this schema, the patient showed a clinically significant benefit and radiological partial response for more than 1 year. Finally, a pulmonary progression was detected, and the patient died in November 2013.

## Discussion

The behaviour of thymic carcinomas is more aggressive than thymomas, with a 5-year overall survival around 40% [3–4]. The recommended first-line treatment for advanced stages is combination chemotherapy, usually platinum-based schemes, with the most common being CAP, ADOC (cisplatin, doxorubicin, vincristine, and cyclophosphamide) and carboplatin-paclitaxel regimes, and an overall response rate ranging between 30% and 50% and a median progression-free interval of approximately 6 months [6–8]. Unfortunately, the evidence of the benefit of treatment with successive lines is scarce, mainly based on studies with a few patients or an isolated case report. There are small amounts of data published using chemotherapeutic drugs (ifosfamide, cisplatin, etoposide, irinotecan, or pemetrexed) and other agents (octreotide with or without prednisone, belinostat, erlotinib, gefinitib, imatinib, sacaratinib, sorafenib, sunitinib, or cetuximab) [9–11].

Metronomic treatment is the chronic administration of chemotherapy at low, minimally toxic doses on a frequent schedule of administration, with no prolonged drug-free breaks [12]. Although the mechanism of action of metronomic chemotherapy is not fully defined, antiangiogenic and antitumor effects—by restoration of the immune system—have been postulated [12, 14]. In this particular area, Kivrak *et al* [13] recently published a case report showing a complete response in a patient with recurrent thymic carcinoma treated with cyclophosphamide and etoposide in a metronomic strategy. Moreover, André *et al* proposed a new strategy based on the combination of metronomic chemotherapy and drug reposition named metronomics [15, 16]. In our patient, this metronomics approach was successful, offering to the patient a relevant clinical benefit with low toxicity and a partial radiological response in his seventh line of treatment, being the only objective response since 2005. In our experience, the metronomics approach deserves further investigation in this subset of patients.

## Conclusions

The interest of this case lies in the response to the therapy used, taking into account that it was a thymic carcinoma with an evolution of 11 years, and that all components of the scheme had already been used in previous lines, but not using a metronomic approach. To our knowledge, there are no published studies using this combination in such as advanced line. The good tolerability and the advantages of the oral administration suggest that this combination should be further analysed.

## Conflicts of interest

The authors have no conflicts of interest to declare.
